# What Drives Metal Artifacts in CBCT? A Comparative Study of Detector Types and Metallic Object Configurations

**DOI:** 10.1155/rrp/7326369

**Published:** 2026-06-07

**Authors:** Zahra Vasegh, Yaser Safi, Mina Iranparvar Alamdari, Katayoun Ghaffari, Mehdi Hosseinzadeh

**Affiliations:** ^1^ Department of Oral and Maxillofacial Radiology, School of Dentistry, Shahid Beheshti University of Medical Sciences, Tehran, Iran, sbmu.ac.ir; ^2^ Department of Oral and Maxillofacial Radiology, School of Dentistry, AJA University of Medical Sciences, Tehran, Iran, ajaums.ac.ir; ^3^ Department of Oral and Maxillofacial Radiology, School of Dentistry, Ardabil University of Medical Sciences, Ardabil, Iran, arums.ac.ir

**Keywords:** amalgam, artifacts, cone beam computed tomography, dental implants, titanium

## Abstract

**Background:**

One major limitation of cone beam computed tomography (CBCT) systems is the presence of metallic artifacts. Given the widespread use of metals in dental treatments and the diversity of CBCT designs, this study aimed to compare the effect of aligned and lateral offset detectors on metal artifact formation within the field of view (FOV).

**Methods:**

This in vitro cross‐sectional study evaluated four CBCT systems—two with aligned and two with lateral offset detectors. A dry human skull phantom was scanned with various metallic objects (a titanium implant, a tooth with a chrome‐cobalt post and PFM crown, and a tooth with an amalgam restoration) placed in the FOV in two orientations (same vs. opposite side of the region of interest [ROI]) and in three quantities (1, 2, or 3 objects). A polypropylene tube filled with dipotassium phosphate solution was placed in the right first premolar socket to simulate alveolar bone. Ten axial slices were analyzed using 1.5‐mm circular ROIs. Mean gray value (MGV) and standard deviation (SD) were calculated, where higher MGV and lower SD indicate lower artifact severity. Data were analyzed with four‐way weighted analysis of variance (ANOVA), and multiple comparisons were performed using Games–Howell, Bonferroni, and *t*‐tests (*p* < 0.05).

**Results:**

Detector type, material, object location, and number significantly affected artifact formation. Aligned detectors showed significantly higher MGV and lower SD, indicating reduced artifact severity (*p* < 0.001). Amalgam produced the most severe artifacts, reflected by the lowest MGVs, while cobalt‐chromium yielded the highest variability (SD) (*p* < 0.005). Artifacts were more severe when metallic objects were on the same side as the ROI and increased with more objects. Several significant interaction effects were observed (*p* < 0.05).

**Conclusion:**

Detector type, metal composition, and the number and position of metallic objects within the FOV significantly influence artifact formation in CBCT images. Optimizing these factors can reduce artifacts and improve image quality.

## 1. Introduction

Dental radiographs are essential tools in dental examinations and treatments. Depending on the clinical need, various imaging techniques are employed. With the increasing use of dental implants for replacing missing teeth, precise evaluation of the surgical site is crucial to minimize intraoperative and postoperative complications [1]. High‐quality imaging enhances treatment success, making cone beam computed tomography (CBCT) a preferred modality in dentistry due to its ability to provide three‐dimensional (3D) reconstructions of maxillofacial structures [2, 3].

CBCT has gained widespread adoption in dentistry due to its advantages over conventional computed tomography (CT), including compact device size, lower cost, high scanning speed, submillimeter resolution, reduced radiation dose, isotropic voxels, accurate image dimensions, and advanced reconstruction software [4, 5]. It is widely used in implantology, oral and maxillofacial surgery, endodontics, and orthodontics for diagnosis, treatment planning, and follow‐up. However, despite these advantages, certain factors can compromise CBCT image quality and hinder accurate interpretation [6].

A key limitation of CBCT imaging is the presence of artifacts, which degrade image quality. Among these, beam hardening artifacts—caused by high‐density materials such as titanium implants, amalgam restorations, and metal prostheses—pose significant challenges. These artifacts reduce contrast, obscure anatomical structures, and complicate diagnosis. The composition, quantity, and positioning of metal objects directly influence CBCT image quality [7].

In recent years, the use of a small field of view (FOV) has gained popularity due to its ability to improve image quality and reduce radiation exposure [8]. The FOV in CBCT imaging depends on factors such as detector size, shape, and radiation geometry. For imaging regions larger than the FOV, two methods are commonly employed [9]:

• Stitching method: Combines multiple scans but increases radiation exposure in overlapping areas.
• Lateral offset method: Uses asymmetric collimation to capture half of the region of interest (ROI) per scan.

In CBCT systems with aligned detectors, the X‐ray beam is symmetrically centered on the detector, and the central ray passes through the center of both the detector and the ROI. In contrast, in systems using a lateral offset detector configuration, the detector is laterally shifted relative to the central ray, and the X‐ray beam is asymmetrically collimated to extend the effective imaging area beyond the detector’s physical center. This configuration allows imaging of regions outside the detector’s central axis but may alter the spatial distribution of artifacts [9].

Although extensive research has examined FOV size and its impact on artifacts, the influence of detector type on metal‐induced artifacts remains unexplored. Given the widespread use of metallic materials in dentistry and the diversity of CBCT detectors, this study aims to investigate the effect of aligned and lateral offset detector types on metal‐induced artifacts within the FOV in CBCT imaging.

## 2. Materials and Methods

### 2.1. Sample Size

This study involved four independent factors: detector type (two types), material type (three types), side (two sides), and number of metallic objects (three levels), resulting in 36 possible experimental conditions. Since no closed mathematical formula was available for sample size determination, a power analysis was conducted using the analysis of variance (ANOVA) module in SPSS (Version 2021). Based on a Type I error (α) of 5%, a Type II error (β) of 20% (corresponding to a power of 80%), and an effect size of 0.2, the required sample size per condition was determined to be 20, leading to a total sample size of 720 (Table 1).

**TABLE 1:** Distribution of sample size across detector types, metallic materials, sides, and number of metallic objects.

Variable		Sample size
Detector	Aligned	360
	Lateral offset	360

Material	Amalgam build‐up	240
	PFM with Cr‐Co post	240
	Titanium implant	240

Side	Left	360
	Right	360

Number of objects	1	240
	2	240
	3	240

### 2.2. Study Design

This in vitro descriptive–analytical cross‐sectional study was conducted using CBCT scans obtained from four different devices.

### 2.3. CBCT Devices and Settings

• Aligned detector:
  ◦ *NewTom VGi* (Verona, Italy): 110 kVp, automatic mA, FOV 8 × 8 cm, voxel size 150 µm
  ◦ *NewTom Giano* (Verona, Italy): 90 kVp, 6 mA, FOV 8 × 8 cm, voxel size 150 µm
• Lateral offset detector:
  ◦ *Carestream CS8200* (Kodak, France): 90 kVp, 6 mA, FOV 8 × 5 cm, voxel size 150 µm
  ◦ *LargeV Smart3D-X* (China): 90 kVp, 6 mA, FOV 8 × 8 cm, voxel size 150 µm

### 2.4. Phantom and Experimental Setup

CBCT imaging was performed on a dry human maxilla containing three types of metallic objects placed within the FOV:

1. Titanium implant (4.8 × 10 mm; Straumann, Switzerland)
2. Chrome‐cobalt post with PFM crown (Neodontics, Inc., USA)
3. Amalgam buildup (SDI, Victoria, Australia)

**FIGURE 1:**
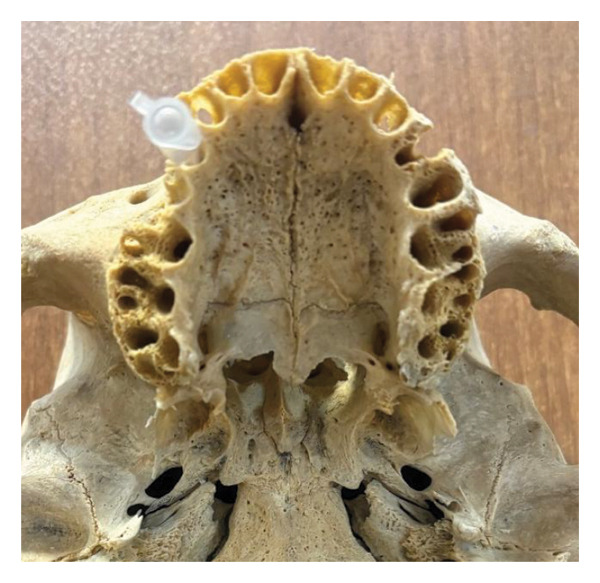
Dipotassium phosphate (K_2_HPO_4_) solution in a polypropylene positioned in the socket of the first premolar on the right side of the maxilla.

To simulate alveolar bone density, a homogeneous dipotassium phosphate (K_2_HPO_4_) solution (1000 mg/mL) was prepared and placed in a polypropylene tube (0.2 mL volume, 15 mm height, 5 mm diameter). The tube was positioned in the socket of the first premolar (#4) on the right side of the maxilla (Figure 1).

### 2.5. Metallic Object Placement Configurations

The metallic objects were arranged in six configurations within the maxillary sockets:

1. Single metallic object in the socket of tooth #6 (right side)
2. Two metallic objects in the sockets of teeth #6 and #7 (right side)
3. Three metallic objects in the sockets of teeth #6, #7, and #2 (right side)
4. Single metallic object in the socket of tooth #6 (left side)
5. Two metallic objects in the sockets of teeth #6 and #7 (left side)
6. Three metallic objects in the sockets of teeth #6, #7, and #2 (left side)

To simulate soft tissue, the dry human maxilla was placed in a PVC plastic container (25 × 15 × 6 cm) filled with water. The positioning of metal objects (such as titanium implants) in the maxilla is illustrated in Figure 2.

FIGURE 2Positioning of metal objects in the dry human maxilla. Titanium implants in the socket of the right maxillary first molar (a), right maxillary first and second molars (b), right maxillary first and second molars and lateral incisor (c), left maxillary first molar (d), left maxillary first and second molars (e), and left maxillary first and second molars and lateral incisor (f) in a dry human maxilla.

**Figure:**
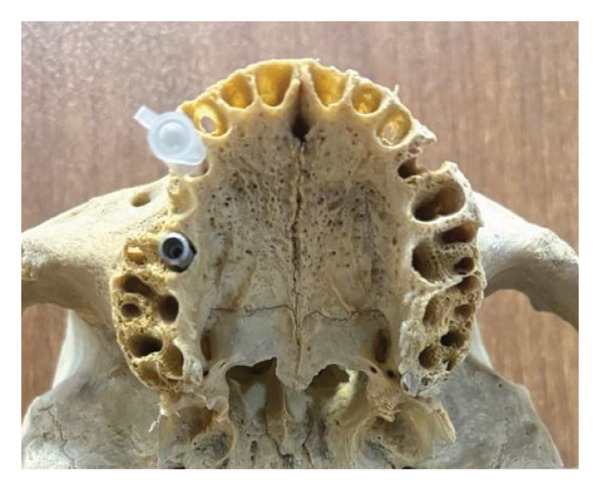
(a)

**Figure:**
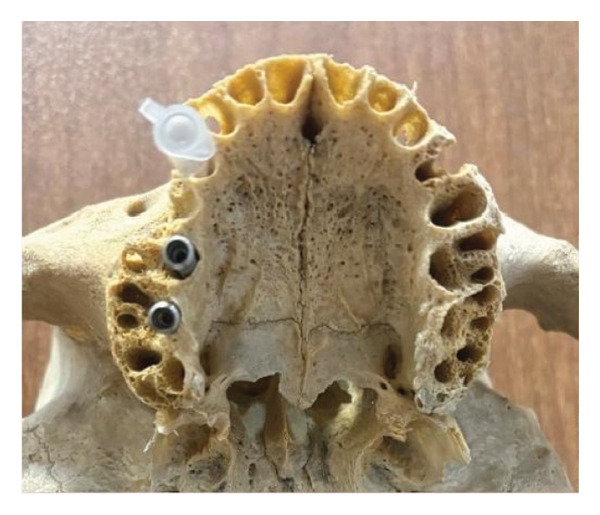
(b)

**Figure:**
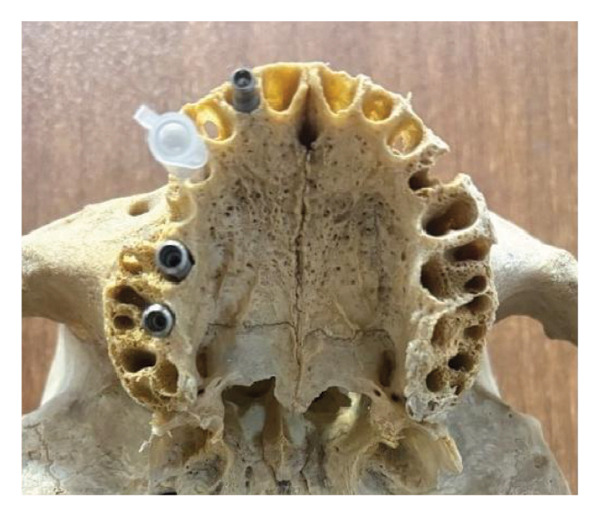
(c)

**Figure:**
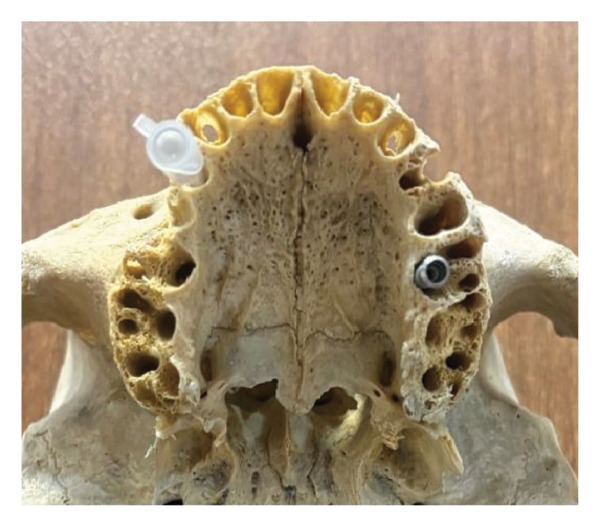
(d)

**Figure:**
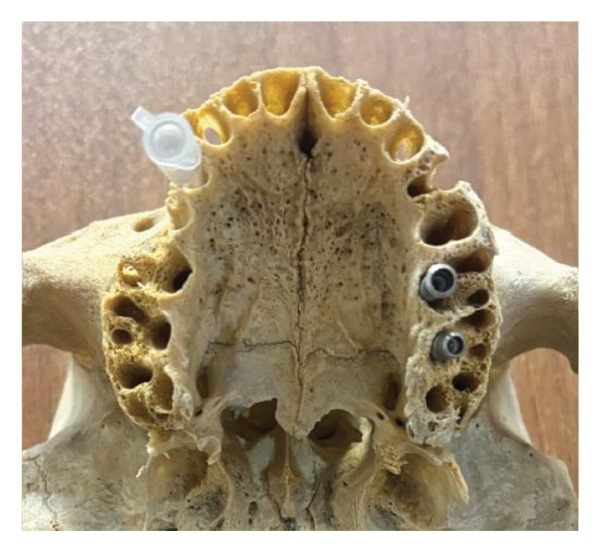
(e)

**Figure:**
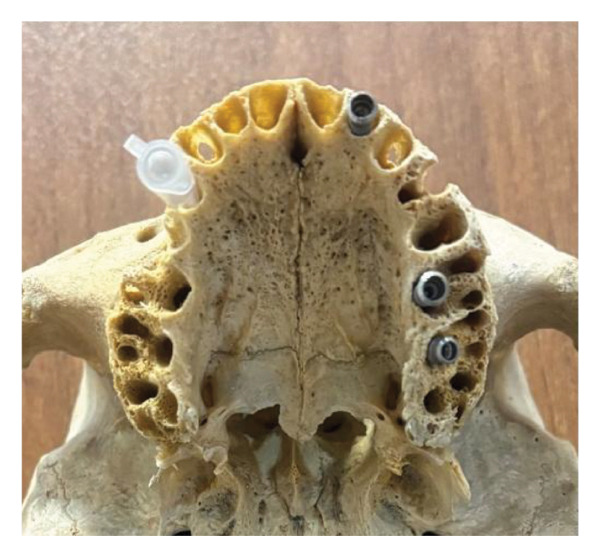
(f)

A control scan (without metallic objects) was performed for each CBCT device.

### 2.6. Image Analysis

A total of 72 CBCT scans were obtained and analyzed using *OnDemand* software (Version 10.0.1). To quantify metal artifacts, an ROI with a 1.5‐mm diameter was defined at the center of the dipotassium phosphate tube in the axial plane. Because CBCT gray values are not standardized and vary depending on device characteristics and reconstruction algorithms, mean gray value (MGV) was used in this study as a relative metric for intrastudy comparisons under controlled imaging conditions, rather than as an absolute quantitative measure. The following gray value parameters were measured:

• Maximum and minimum values
• Mean and standard deviation (SD)

These measurements were extracted from 10 consecutive axial slices (1‐mm intervals). The most coronal slice was 1 mm apical to the uppermost part of the dipotassium phosphate material, while the most apical slice corresponded to the apex of the tube. From these 10 slices per scan, repeated measurements were collected under identical imaging conditions, resulting in a total of 720 observations used for weighted statistical analysis. The methodology for measuring gray values (such as titanium implant) in scans is illustrated in Figure 3.

FIGURE 3Methodology for measuring gray values in CBCT scans. Titanium implants in the socket of the right maxillary first molar (a), right maxillary first and second molars (b), right maxillary first and second molars and lateral incisor (c), left maxillary first molar (d), left maxillary first and second molars (e), and left maxillary first and second molars and lateral incisor (f) in a dry human maxilla.

**Figure:**
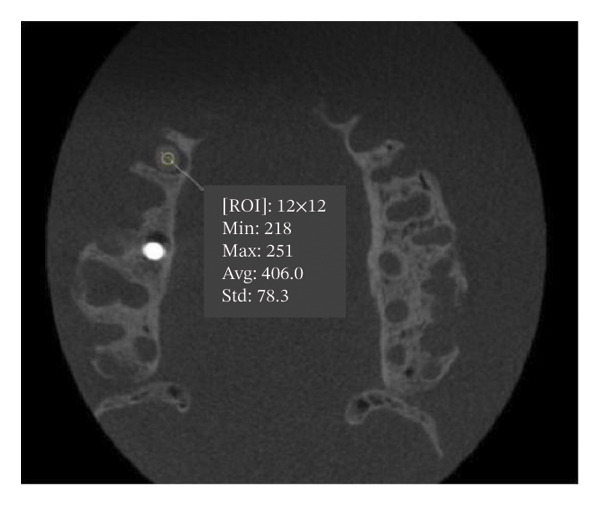
(a)

**Figure:**
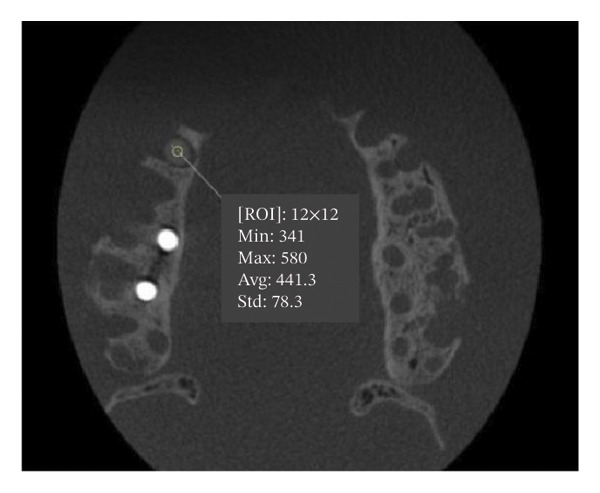
(b)

**Figure:**
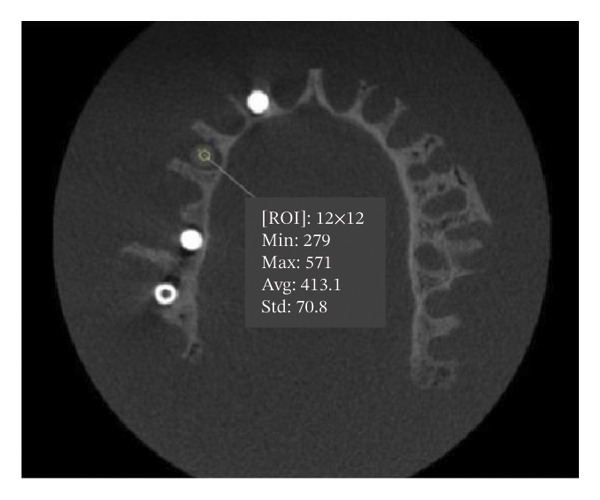
(c)

**Figure:**
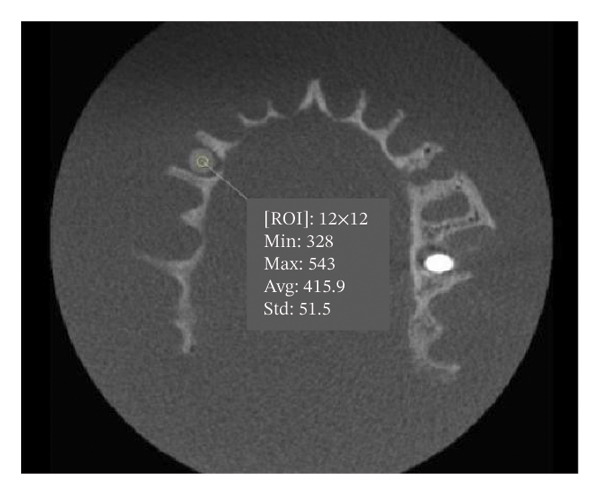
(d)

**Figure:**
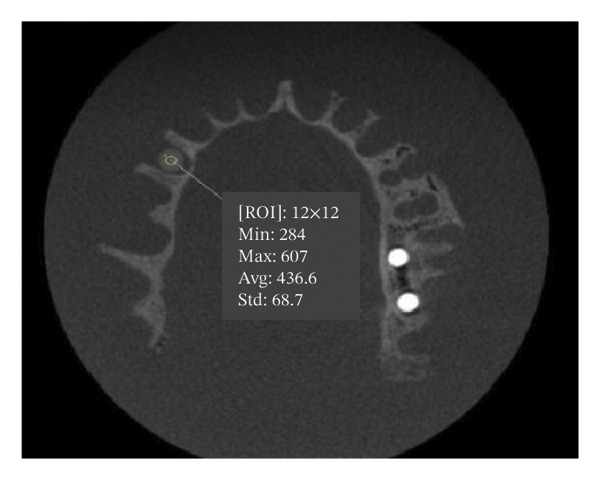
(e)

**Figure:**
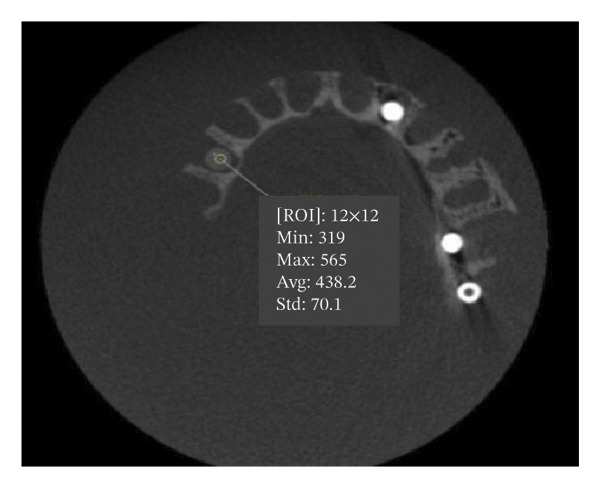
(f)

### 2.7. Statistical Analysis

After determining the required sample size, the relevant data were recorded and measured. Following data collection, statistical analysis and graph plotting were performed using SPSS 26. The Shapiro–Wilk test was used to assess the normality of data distribution, and Levene’s test was employed to examine the equality of variances. A weighted four‐way ANOVA was conducted to evaluate the main and interaction effects of four factors: material, detector, direction, and quantity. Because multiple slice measurements were obtained from each CBCT scan, the data had a clustered structure, with slices nested within scans. To address this, a weighted statistical analysis was used to account for the contribution of repeated measurements from the same scan and to avoid overrepresentation of individual scans in the analysis. MGV and SD measurements from consecutive slices were treated as repeated observations within each scan under consistent imaging conditions, and weighting was applied to ensure balanced representation across experimental conditions. Additionally, the Games–Howell and Bonferroni post hoc tests were used for pairwise comparisons, while the independent samples *t*‐test was applied to compare the means of two independent groups.

### 2.8. Ethical Considerations

This study was conducted in vitro using CBCT scans of a phantom; thus, no ethical approval or informed consent was required. Radiation protection protocols were followed throughout the study. The study protocol was approved by the university’s ethics committee (number: IR.SBMU.DRC.REC.1403.038).

## 3. Results

This study evaluated artifact formation in CBCT images from two perspectives: (1) MGVs and (2) the SD of gray values.

### 3.1. Effect of Detector Type

The MGV, where higher values indicate lower artifact severity, was significantly higher in the aligned detector compared to the lateral offset detector (*p* = 0.001).

This indicates that, based on MGV, the artifact generated by the lateral offset detector is greater than that of the aligned detector.

Moreover, the SD was significantly lower in the aligned detector group compared to the lateral offset detector group (*p* = 0.001), suggesting that the artifact intensity is more consistent and less pronounced in the aligned detector (Table 2).

**TABLE 2:** Bonferroni multiple comparisons of detector type, material, side, and number of objects based on gray value mean and standard deviation.

Variable	Group I	Group J	Mean gray value			Standard deviation		
			Mean difference	Standard error	*p* value	Mean difference	Standard error	*p* value
Detector	Aligned	Lateral offset	12.464	2.373	0.001	−13.125	2.322	0.001

Material	Amalgam build‐up	PFM with Cr‐Co post	−41.980	24.639	0.267	−0.273	2.671	1.000
		Titanium implant	−68.515	21.198	0.004	14.532	2.373	0.001
	PFM with Cr‐Co post	Titanium implant	−26.535	22.351	0.707	14.805	2.408	0.001

Side	Left	Right	10.642	2.537	0.011	−10.211	2.596	0.001

Number of objects	1	2	55.617	21.691	0.032	−2.664	2.316	0.751
		3	27.469	22.115	0.644	−13.364	2.537	0.001
	2	3	−28.147	23.445	0.691	−10.700	2.596	0.001

*Note:* Mean difference represents the difference between Group I and Group J (Group I − Group J). Positive values indicate higher mean gray values in Group I, while negative values indicate higher values in Group J.

### 3.2. Effect of Material Type

Regarding the type of metallic object, the MGV followed this order: amalgam < cobalt‐chromium < titanium, indicating greater artifact severity in amalgam and lower artifact severity in titanium. However, a statistically significant difference was only observed between amalgam and titanium (*p* = 0.004), indicating that, based on MGV, amalgam produced greater artifacts.

The SD followed this order: titanium < amalgam < cobalt‐chromium. Statistically significant differences were observed between titanium and amalgam (*p* = 0.001), as well as between titanium and cobalt‐chromium (*p* = 0.001). These findings suggest that, based on SD, cobalt‐chromium and amalgam generate more artifacts than titanium (Table 2).

### 3.3. Effect of Side

The MGV was significantly higher when the metal object was placed in the opposite side (left) compared to the same side (right) (*p* = 0.001), indicating reduced artifact severity in that configuration. This implies that, based on MGV, artifacts were less severe in the opposite orientation.

The SD was significantly higher on the same side compared to the opposite side (*p* = 0.001), indicating that the same side resulted in greater artifact variability and severity (Table 2).

### 3.4. Effect of the Number of Metallic Objects

The comparison of MGV across different numbers of metal objects revealed the following order: 2 objects < 3 objects < 1 object, indicating that artifact severity was lowest in the single‐object configuration. A statistically significant difference was found only between the one‐object and two‐object groups (*p* = 0.032), suggesting that a single metal object produced the least amount of artifact based on MGV.

For SD, the values increased progressively with the number of objects: 1 object < 2 objects < 3 objects, indicating a correlation between the number of metal objects and artifact severity (Table 2).

### 3.5. Interaction Effects Between Variables

Significant interaction effects were observed for certain variable combinations:

#### 3.5.1. MGV

##### 3.5.1.1. Metallic Object Type and Placement Side

**FIGURE 4:**
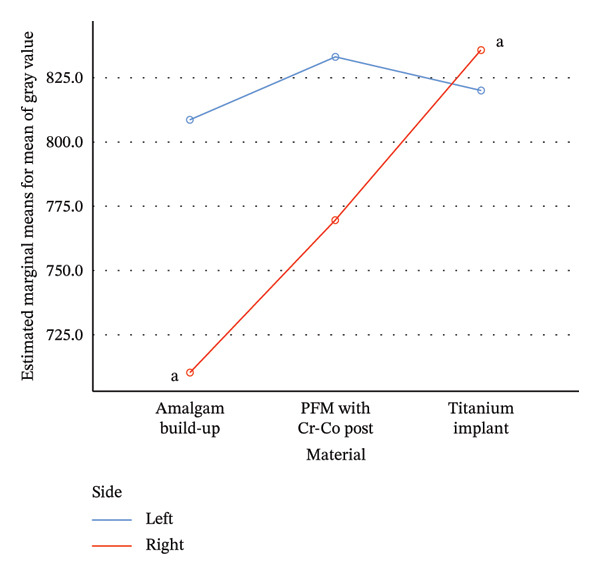
Interaction effects between variables based on mean gray value (MGV). Higher MGVs indicate lower artifact severity and improved image quality, whereas lower MGVs indicate increased artifact severity. Different letters indicate statistically significant differences between groups (Games–Howell post hoc test, *p* < 0.05). Pairwise comparison: a, *p* = 0.018.

When the metallic objects were positioned on the left side, the MGV followed the order of amalgam < titanium < cobalt‐chromium; however, these differences were not statistically significant. In contrast, when the objects were placed on the right side, the MGV followed the order of amalgam < cobalt‐chromium < titanium. In this configuration, the difference between amalgam and titanium was statistically significant (Table 3) (Figure 4).

**TABLE 3:** Games–Howell multiple comparisons for the interaction between side and material based on gray value mean and standard deviation.

Variables			Mean		SD	
Side	(I) Material	(J) Material	Standard error	*p* value	Standard error	*p* value
Left	Amalgam build‐up	PFM with Cr‐Co post	36.9290	0.989	3.6018	0.871
		Titanium implant	34.1156	0.894	3.2839	0.035
	PFM with Cr‐Co post	Titanium implant	36.8154	0.959	3.5092	0.013

Right	Amalgam build‐up	PFM with Cr‐Co post	34.0121	0.997	4.8939	0.652
		Titanium implant	32.9775	0.018	4.2783	0.003
	PFM with Cr‐Co post	Titanium implant	37.9448	0.055	4.0348	0.001

#### 3.5.2. SD

**FIGURE 5:**
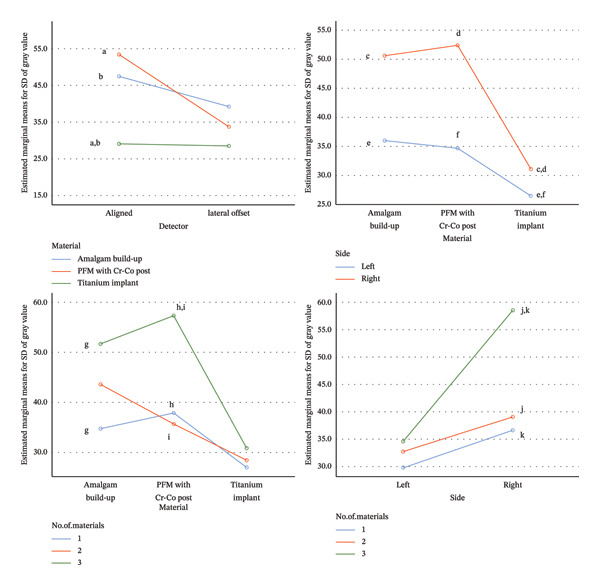
Interaction effects between variables based on the standard deviation (SD) of gray values. Higher SD values indicate greater artifact severity and increased gray value variability, whereas lower SD values indicate reduced artifact severity and more homogeneous image quality. Different letters indicate statistically significant differences between groups (Games–Howell post hoc test, *p* < 0.05). Pairwise comparisons: a, *p* = 0.001; b, *p* = 0.001; c, *p* = 0.003; d, *p* = 0.001; e, *p* = 0.035; f, *p* = 0.013; g, *p* = 0.010; h, *p* = 0.014; i, *p* = 0.026; j, *p* = 0.009; k, *p* = 0.002.

1. Detector and material: In the aligned detector, the SD of gray values followed the order: titanium < amalgam < cobalt‐chromium. Statistically significant differences were observed between amalgam and titanium, as well as between cobalt‐chromium and titanium. In the lateral offset detector, the SD followed the order: titanium < cobalt‐chromium < amalgam; however, these differences were not statistically significant (Table 4) (Figure 5).
2. Material and side: When the metallic objects were placed on the left side, the SD of gray values followed the order: titanium < cobalt‐chromium < amalgam. When placed on the right side, the order was titanium < amalgam < cobalt‐chromium. In both orientations, the differences between amalgam and titanium, as well as between cobalt‐chromium and titanium, were statistically significant (Table 3) (Figure 5).
3. Material and number of objects: For amalgam, the SD of gray values followed the order: 3 objects > 2 objects > 1 object, with a statistically significant difference observed only between one and three objects. In the case of cobalt‐chromium, the order was 3 objects > 1 object > 2 objects, with significant differences between one and three objects, as well as between two and three objects. For titanium, the SD followed the order: 3 objects > 2 objects > 1 object; however, none of the differences among the groups were statistically significant (Table 5) (Figure 5).
4. Number of objects and placement side: When metallic objects were placed on both the left and right sides, the SD of gray values followed the same trend: 3 objects > 2 objects > 1 object. On the left side, none of the differences among the groups were statistically significant. However, on the right side, significant differences were observed between one and three objects, as well as between two and three objects (Table 6) (Figure 5).
 No significant interactions were found between:
5. Detector and side.
6. Detector and number of objects.

**TABLE 4:** Games–Howell multiple comparisons for the interaction between detector and material based on gray value standard deviation.

Detector	Material		Standard error	*p* value
Aligned	Amalgam build‐up	PFM with Cr‐Co post	3.4091	0.253
		Titanium implant	3.0005	0.001
	PFM with Cr‐Co post	Titanium implant	3.0684	0.001

Lateral offset	Amalgam build‐up	PFM with Cr‐Co post	5.1331	0.990
		Titanium implant	4.5302	0.168
	PFM with Cr‐Co post	Titanium implant	4.4431	0.113

**TABLE 5:** Games–Howell multiple comparisons for the interaction between material and number of objects based on gray value standard deviation.

Material	Number of objects		Standard error	*p* value
Amalgam build‐up	1	2	4.0092	0.403
		3	5.7320	0.010
	2	3	5.8213	0.110

PFM with Cr‐Co post	1	2	4.7095	0.971
		3	5.4262	0.014
	2	3	5.4895	0.026

Titanium implant	1	2	3.9588	0.983
		3	4.0502	0.974
	2	3	3.9001	0.999

**TABLE 6:** Games–Howell multiple comparisons for the interaction between side and number of objects based on gray value standard deviation.

Side	Number of objects		Standard error	*p* value
Left	1	2	3.4302	0.927
		3	3.5254	0.470
	2	3	3.5612	0.701

Right	1	2	3.5206	0.770
		3	4.8166	0.002
	2	3	4.8251	0.009

## 4. Discussion

Given the widespread use of metallic restorations and implants in dentistry, this study aimed to evaluate the influence of detector type—aligned versus lateral offset—on the extent of metal artifacts within the FOV in CBCT imaging, with the goal of optimizing image quality for clinical decision‐making.

While prior research has assessed factors like the type, number, and position of metal objects and exposure settings affecting CBCT artifacts, the role of detector type remains underexplored [10, 11]. Vasegh et al. [12] investigated artifacts using two detector types, but their study focused on metallic objects positioned outside the FOV (exomass).

Most prior studies on metal artifacts were laboratory‐based, using metal objects of uniform size and volume, which does not reflect the diverse clinical applications of metals in dentistry [11, 13, 14]. To better simulate clinical reality, this study uniquely evaluated metal artifacts within the FOV using a dry human maxilla model, providing more clinically relevant conditions.

Despite numerous studies on CBCT metal artifacts, there is no consensus on standardized quantitative measures for their impact on image quality [15]. Most research uses MGV and SD of voxel values, where higher MGV and lower SD indicate fewer artifacts. It should be noted that CBCT gray values are not standardized and are influenced by device‐specific factors, including detector characteristics, reconstruction algorithms, and scatter correction methods. Therefore, MGV should be interpreted as a relative indicator within the same study and imaging conditions, rather than an absolute quantitative parameter. Differences in reconstruction processes may affect gray value distribution independently of artifact severity, and this should be considered when comparing results across different CBCT systems. This study assessed both parameters, aligning with Vazconcelos et al., who identified higher SD as indicative of greater artifact severity [16].

In this study, FOV, kVp, mA, and voxel size were kept constant. An 8 × 8 cm FOV was used for most devices, with one using 8 × 5 cm. Smaller FOVs are preferred to reduce patient radiation dose and scatter, thereby improving image quality [8, 17, 18]. Consistent with prior research, using the smallest FOV encompassing all metallic objects helped minimize noise and gray value variability. Accurate selection of exposure parameters is essential for balancing image quality and radiation dose in CBCT imaging. Increasing tube voltage (kVp) increases the energy and penetration capability of the X‐ray beam, which reduces beam hardening effects and image noise, resulting in more homogeneous images. However, the relationship between kVp and patient dose is not linear and depends on the combination of exposure parameters, including mA, exposure time, and device‐specific optimization protocols. In some cases, increasing kVp allows for reduction of tube current or exposure time while maintaining diagnostic image quality, which may result in an overall reduction in radiation dose. Oliveira et al. [14] reported that kVp has a greater influence than mA on artifact expression, with higher kVp producing more homogeneous images and reducing artifact severity. Therefore, kVp should be considered as part of an optimized exposure protocol rather than an independent determinant of radiation dose. This principle is consistent with the ALARA concept, in which exposure parameters are adjusted collectively to achieve adequate image quality with the lowest possible radiation dose. Voxel size significantly affects CBCT image quality and metal artifacts, with smaller voxels enhancing detail but potentially increasing artifacts, while larger voxels reduce artifacts at the expense of accuracy [19–21]. This study used a 0.15‐mm voxel size as the standard resolution across all devices to balance these factors.

Another important consideration is the potential influence of system‐specific technical parameters on artifact expression. Reconstruction algorithms vary among CBCT systems and may significantly affect artifact severity, as differences in beam hardening correction, scatter reduction, and image processing techniques can influence gray value distribution and image noise. These proprietary reconstruction processes are typically not fully disclosed by manufacturers, making direct comparison challenging. Additionally, imaging parameters such as FOV size and exposure settings, including tube voltage (kVp), may also influence artifact formation. In the present study, a standardized FOV (8 × 5 cm) and exposure parameters were selected based on manufacturer recommendations and clinical relevance; however, these settings may interact with detector configuration and system design. Higher kVp settings may reduce beam hardening effects due to increased photon energy, while FOV size can influence scatter radiation and artifact distribution. Therefore, the observed differences in artifact expression likely reflect the combined influence of detector configuration and system‐specific acquisition and reconstruction characteristics rather than detector geometry alone.

This study evaluated detector type, metal type, side of material, and number of metallic objects.

### 4.1. Impact of Detector Type

In the present study, metallic artifacts were evaluated using two types of detectors. Based on MGV and SD, CBCT systems equipped with aligned detectors demonstrated lower artifact levels compared to systems using lateral offset detectors under the evaluated imaging conditions. Santaella et al. [22] also compared aligned and lateral offset detectors, reporting that artifact reduction algorithms performed more effectively with aligned detectors. Although the current study did not assess the performance of metal artifact reduction (MAR) algorithms, their impact could have been a relevant factor. In another study, Santaella et al. [23] examined MGV and SD across different FOV regions and found that lateral offset detectors exhibited more variability in SD compared to aligned detectors. While SD was similar between the two groups in our study, the aligned detector yielded significantly higher MGVs, indicating reduced artifact levels. These findings are consistent with previous research, including the study by Vasegh et al. [12], which also showed that aligned detectors produce fewer artifacts than lateral offset detectors. Given the limited number of studies comparing these detector types, this investigation was deemed necessary.

### 4.2. Effect of Metal Type

The present study demonstrated that titanium implants produced the least and amalgam restorations the highest level of artifacts in CBCT images. These findings align with previous studies by Codari et al. [19] and Bălută et al. [24], which reported that high‐density materials such as amalgam and cobalt‐chromium generate more pronounced metallic artifacts. This phenomenon is likely due to differences in physical and atomic properties, as materials with higher density and atomic number cause greater beam hardening, leading to dark and bright streaks in CBCT images. Kuusisto et al. [25] also showed that increased radiopacity intensifies metallic artifacts, suggesting a threshold effect at 20% radiopaque content.

Similarly, Safi et al. [26] reported that the type of metal object located in the exomass region significantly affected artifact severity, with the highest artifact levels seen in amalgam, followed by cobalt‐chromium and titanium—consistent with the present study. However, Safi et al. [26] evaluated only one CBCT unit, whereas the current study included four different devices.

Demirturk et al. [27] compared titanium and zirconia implants and found that zirconia generated more artifacts. In our study, zirconia was not included due to its lower clinical prevalence, and titanium implants consistently showed the least artifact based on both MGV and SD metrics.

In contrast, Vasegh et al. [12] found no statistically significant differences in artifact severity among various metals, despite cobalt‐chromium showing the highest values. They reported equal MGVs among all metal samples and the control group, attributing the outcome to differences in metal volume. Notably, their study used the same metal materials (in terms of size and brand) as ours. Two factors may explain the discrepancy: (1) in Vasegh’s study, metal objects were placed as exomass, and (2) they were located farther from the ROI.

### 4.3. Effect of Side

In addition to the type and composition of metal objects, their spatial orientation significantly influences artifact severity. In the present study, metal objects positioned on the same side as the ROI generated more pronounced artifacts—evidenced by lower MGVs and higher SD—compared to those located on the opposite side. This finding is consistent with the study by Martins et al. [28], which had a comparable imaging protocol and showed that metal objects closer to the ROI produce greater artifacts. However, their study did not specifically assess the statistical significance of artifact differences based on directional alignment with the ROI.

Our results are also in line with those of Demirturk et al. [27], who reported that the spatial position of metal objects markedly affects image distortion. They found that exomass metal objects caused more artifacts. While it is generally accepted that metals within the FOV produce more artifacts, their study indicated that under certain conditions—such as small FOV size, use of MAR algorithms, and the presence of high atomic number materials like zirconia—exomass metals can produce greater artifacts. This underscores the importance of carefully considering imaging parameters and clinical context in CBCT, particularly when high‐density materials are present.

Similarly, Vasegh et al. [12] reported that the highest artifact levels (based on MGV and SD) occurred when metal objects were placed on both sides of the ROI, and the lowest occurred when objects were located on the opposite side of the ROI. Additionally, Santaella et al. [23] found that artifact levels tend to be higher in peripheral FOV regions. Băluță et al. [24] also observed increased artifact formation in the anterior regions and on the lingual side of metal objects.

### 4.4. Effect of the Number of Metal Objects

Another key variable examined in this study was the number of metal objects present within the FOV. The results revealed that an increase in the number of metallic objects corresponded with a greater degree of metal artifacts. Previous studies have similarly reported that a higher number of metallic items, particularly within the exomass region, lead to decreased MGVs and increased gray value SDs [26, 29, 30].

These findings align with those of Martins et al. [28], Vasegh et al. [12], and Safi et al. [26], all of whom concluded that the presence of multiple implants—either within the FOV or in the exomass—intensifies artifacts and degrades image quality. This phenomenon is likely due to increased X‐ray beam interference and more pronounced beam hardening in the presence of multiple dense materials.

Several approaches have been explored to reduce metal artifacts in CBCT imaging. These include the use of small FOVs, antiscatter grids, and adjustment of exposure parameters such as kVp and mA, each demonstrating varying degrees of success [31]. Additionally, software‐based MAR algorithms have shown promise in minimizing artifact severity [32]. However, none of these strategies has been entirely effective in eliminating metal artifacts. For instance, Martins et al. [28] found that MAR performance depends on implant positioning, while Candemil et al. [9] reported that MAR algorithms in devices like PicassoTrio and ProMax are only effective when the metallic objects are located within the FOV.

It should also be noted that differences between CBCT systems extend beyond detector alignment and include variations in exposure parameters, detector electronics, and proprietary reconstruction algorithms. These factors may influence image noise characteristics and artifact expression. Therefore, while detector alignment appears to be an important contributing factor, the results of this study should be interpreted within the context of the specific CBCT systems evaluated rather than generalized solely to detector geometry.

### 4.5. Limitation

This study has several limitations that should be acknowledged. Although detector alignment was the primary variable of interest, each detector type was represented by different CBCT systems, which inherently differed in technical characteristics, including tube voltage, mA control mode (fixed versus automatic), FOV dimensions, and manufacturer‐specific reconstruction algorithms. These system‐dependent factors may have influenced artifact expression and therefore represent potential confounding variables. In particular, automatic mA modulation adjusts tube current according to object attenuation to optimize image quality, which may introduce variability compared with fixed mA settings. As a result, the observed differences cannot be attributed exclusively to detector alignment but rather reflect the combined influence of detector configuration and system‐specific imaging parameters.

The study was conducted in a laboratory setting using a dry human maxilla phantom, which may not fully replicate clinical conditions where soft tissue variability and patient‐related factors can influence artifact formation. Future clinical studies using patient CBCT scans are recommended to improve external validity.

MAR algorithms were not evaluated, and since MAR performance varies across CBCT systems and detector configurations, future research should investigate their interaction with detector alignment and artifact formation.

Finally, although efforts were made to standardize voxel size and maintain clinically relevant exposure settings, subtle differences in acquisition protocols between devices may have affected artifact measurements. Additionally, multiple slice measurements were obtained from each CBCT scan, resulting in clustered observations rather than fully independent data points. Although weighted statistical analysis was applied to reduce potential bias, some degree of intrascan correlation may remain. Future studies may benefit from using scan‐level averaging or advanced statistical models, such as mixed‐effects analysis, to fully account for the hierarchical structure of the data.

## 5. Conclusion

Within the limitations of this in vitro study, CBCT systems with aligned detectors demonstrated lower artifact expression compared with systems using lateral offset detectors under the evaluated imaging conditions. These findings reflect the combined influence of detector configuration and system‐specific technical characteristics, including exposure parameters and reconstruction algorithms, and should not be attributed solely to detector geometry. Artifact severity was also influenced by metallic object type, number, and spatial orientation: amalgam and cobalt‐chromium produced greater artifacts than titanium, and artifact intensity increased with the number of metallic objects and when they were positioned closer to the ROI. These results highlight the importance of considering both detector configuration and system‐specific factors when interpreting CBCT images in the presence of metallic restorations.

## Author Contributions

Zahra Vasegh: conceptualization, methodology, formal analysis, writing–original draft.

Yaser Safi: data curation, investigation, writing–review and editing.

Mina Iranparvar Alamdari: software, validation, visualization.

Katayoun Ghaffari: resources, supervision.

Mehdi Hosseinzadeh: project administration, funding acquisition, supervision, writing–review and editing.

## Funding

No funding was received for this manuscript.

## Disclosure

We confirm that no person or third‐party services were involved in the research or manuscript preparation who are not listed as authors or acknowledged.

## Conflicts of Interest

The authors declare no conflicts of interest.

## Data Availability

The data that support the findings of this study are available from the corresponding author upon reasonable request.

## References

[1] Venkatesh E. and Elluru S. V. , Cone Beam Computed Tomography: Basics and Applications in Dentistry, Journal of Istanbul University Faculty of Dentistry. (2017) 51, no. 3, 102–121. 10.17096/jiufd.00289 PMC5750833 29354314

[2] Bornstein M. M. , Scarfe W. C. , Vaughn V. M. , and Jacobs R. , Cone Beam Computed Tomography in Implant Dentistry: a Systematic Review Focusing on Guidelines, Indications, and Radiation Dose Risks, The International Journal of Oral & Maxillofacial Implants. (2014) 29, no. S1, 55–77, 10.11607/jomi.2014suppl.g1.4. 24660190

[3] Jacobs R. , Salmon B. , Codari M. , Hassan B. , and Bornstein M. M. , Cone Beam Computed Tomography in Implant Dentistry: Recommendations for Clinical Use, BMC Oral Health. (2018) 18, 1–16, 10.1186/s12903-018-0523-5. 29764458 PMC5952365

[4] Ganz S. D. , CT/CBCT Diagnosis and Treatment Planning Concepts for Bone Grafting Applications, Implant Site Development. (2015) 101–120.

[5] Mol A. , Mallya S. , and Lam E. , White and Pharoah’s Oral Radiology: Principles and Interpretation, 2018.

[6] Helvacioglu-Yigit D. , Demirturk Kocasarac H. , Bechara B. , and Noujeim M. , Evaluation and Reduction of Artifacts Generated by 4 Different Root-End Filling Materials by Using Multiple Cone-Beam Computed Tomography Imaging Settings, Journal of Endodontics. (2016) 42, no. 2, 307–314, 10.1016/j.joen.2015.11.002. 26711863

[7] Leng S. , Zambelli J. , Tolakanahalli R. et al., Streaking Artifacts Reduction in Four-Dimensional Cone-Beam Computed Tomography, Medical Physics. (2008) 35, no. 10, 4649–4659, 10.1118/1.2977736. 18975711 PMC2655146

[8] Pauwels R. , Jacobs R. , Bogaerts R. , Bosmans H. , and Panmekiate S. , Reduction of Scatter-Induced Image Noise in Cone Beam Computed Tomography: Effect of Field of View Size and Position, Oral Surgery, Oral Medicine, Oral Pathology and Oral Radiology. (2016) 121, no. 2, 188–195, 10.1016/j.oooo.2015.10.017. 26792756

[9] Candemil A. P. , Salmon B. , Freitas D. Q. , Ambrosano G. M. B. , Haiter-Neto F. , and Oliveira M. L. , Are Metal Artefact Reduction Algorithms Effective to Correct Cone Beam CT Artefacts Arising From the Exomass?, Dentomaxillofacial Radiology. (2019) 48, no. 3, 10.1259/dmfr.20180290. PMC6476351 30540919

[10] Fontenele R. C. , Nascimento E. H. , Vasconcelos T. V. , Noujeim M. , and Freitas D. Q. , Magnitude of Cone Beam CT Image Artifacts Related to Zirconium and Titanium Implants: Impact on Image Quality, Dentomaxillofacial Radiology. (2018) 47, no. 6, 10.1259/dmfr.20180021. PMC6196050 29668300

[11] Candemil A. P. , Salmon B. , Ambrosano G. M. , Freitas D. Q. , Haiter-Neto F. , and Oliveira M. L. , Influence of Voxel Size on Cone Beam Computed Tomography Artifacts Arising From the Exomass, Oral Surgery, Oral Medicine, Oral Pathology and Oral Radiology. (2021) 132, no. 4, 456–464, 10.1016/j.oooo.2020.12.003. 33422474

[12] Vasegh Z. , shirian a , valizadeh s , Safi Y. , and Ghaffari K. , Comparison of the Effect of Aligned and Lateral Offset Detectors on the Artifacts From Metallic Materials in the Exomass in CBCT Imaging, 2024.

[13] Candemil A. P. , Salmon B. , Vasconcelos K. F. et al., Cone Beam CT Optimisation for Detection of Vertical Root Fracture With Metal in the Field of View or the Exomass, Scientific Reports. (2021) 11, no. 1, 10.1038/s41598-021-98345-6. PMC8476605 34580339

[14] Oliveira M. L. , Candemil A. P. , Freitas D. Q. , Haiter-Neto F. , Wenzel A. , and Spin-Neto R. , Objective Assessment of the Combined Effect of Exomass-Related-and Motion Artefacts in Cone Beam CT, Dentomaxillofacial Radiology. (2021) 50, no. 1, 10.1259/dmfr.20200255. PMC7780835 32706986

[15] Fontenele R. C. , Farias Gomes A. , Nejaim Y. , and Freitas D. Q. , Do the Tube Current and Metal Artifact Reduction Influence the Diagnosis of Vertical Root Fracture in a Tooth Positioned in the Vicinity of a Zirconium Implant? A CBCT Study, Clinical Oral Investigations. (2021) 25, no. 4, 2229–2235, 10.1007/s00784-020-03538-4. 32827079

[16] Pauwels R. , Beinsberger J. , Collaert B. et al., Effective Dose Range for Dental Cone Beam Computed Tomography Scanners, European Journal of Radiology. (2012) 81, no. 2, 267–271, 10.1016/j.ejrad.2010.11.028. 21196094

[17] Parsa A. , Ibrahim N. , Hassan B. , van der Stelt P. , and Wismeijer D. , Influence of Object Location in Cone Beam Computed Tomography (NewTom 5G and 3D Accuitomo 170) on Gray Value Measurements at an Implant Site, Oral Radiology. (2014) 30, 153–159.

[18] Yadegari A. , Safi Y. , Shahbazi S. , Yaghoutiazar S. , and Ghazizadeh Ahsaie M. , Assessment of CBCT Gray Value in Different Regions-of-Interest and Fields-of-View Compared to Hounsfield Unit, Dentomaxillofacial Radiology. (2023) 52, no. 8, 10.1259/dmfr.20230187. PMC10968765 37874074

[19] Codari M. , de Faria Vasconcelos K. , Ferreira Pinheiro Nicolielo L. , Haiter Neto F. , and Jacobs R. , Quantitative Evaluation of Metal Artifacts Using Different CBCT Devices, High-Density Materials and Field of Views, Clinical Oral Implants Research. (2017) 28, no. 12, 1509–1514, 10.1111/clr.13019. 28432698

[20] Dong T. , Yuan L. , Liu L. et al., Detection of Alveolar Bone Defects With Three Different Voxel Sizes of Cone-Beam Computed Tomography: An in Vitro Study, Scientific Reports. (2019) 9, no. 1, 10.1038/s41598-019-44675-5. PMC6544761 31148581

[21] Spin-Neto R. , Gotfredsen E. , and Wenzel A. , Impact of Voxel Size Variation on CBCT-Based Diagnostic Outcome in Dentistry: A Systematic Review, Journal of Digital Imaging. (2013) 26, no. 4, 813–820, 10.1007/s10278-012-9562-7. 23254628 PMC3705012

[22] Santaella G. M. , Wenzel A. , Haiter-Neto F. , Rosalen P. L. , and Spin-Neto R. , Impact of Movement and Motion-Artefact Correction on Image Quality and Interpretability in CBCT Units With Aligned and Lateral-Offset Detectors, Dentomaxillofacial Radiology. (2020) 49, no. 1, 10.1259/dmfr.20190240. PMC6957066 31530012

[23] Santaella G. M. , Rosalen P. L. , Queiroz P. M. , Haiter-Neto F. , Wenzel A. , and Spin-Neto R. , Quantitative Assessment of Variation in CBCT Image Technical Parameters Related to CBCT Detector Lateral-Offset Position, Dentomaxillofacial Radiology. (2020) 49, no. 2, 10.1259/dmfr.20190077. PMC7026930 31469317

[24] Băluță A. , Dragomirescu A.-O. , Bencze M.-A. , Vasilache A. , Imre M. , and Ionescu E. , Assessment of The Effects of Tube Current and Voltage Intensity, of The Position and Metal Type on the Occurrence of Metal Artefacts in CBCT Section, Romanian Journal of Oral Rehabilitation. (2021) 13, no. 3.

[25] Kuusisto N. , Vallittu P. K. , Lassila L. V. , and Huumonen S. , Evaluation of Intensity of Artefacts in CBCT by Radio-Opacity of Composite Simulation Models of Implants in Vitro, Dentomaxillofacial Radiology. (2015) 44, no. 2, 10.1259/dmfr.20140157. PMC4614173 25283364

[26] Safi Y. , Ghazizadeh Ahsaie M. , and Jafarian A. M. , Effect of the Field of View Size on CBCT Artifacts Caused by the Presence of Metal Objects in the Exomass, International Journal of Dentistry. (2022) 2022, no. 1, 10.1155/2022/2071108. PMC9481401 36117513

[27] Demirturk K. H. , Koenig L. J. , Ustaoglu G. , Oliveira M. L. , and Freitas D. Q. , CBCT Image Artefacts Generated by Implants Located Inside the Field of View or in the Exomass, Dentomaxillofacial Radiology. (2022) 51, no. 2, 10.1259/dmfr.20210092. PMC8802698 34289314

[28] Martins L. A. C. , Queiroz P. M. , Nejaim Y. , Vasconcelos K. F. , Groppo F. C. , and Haiter-Neto F. , Evaluation of Metal Artefacts for Two CBCT Devices With a New Dental Arch Phantom, Dentomaxillofacial Radiology. (2020) 49, no. 5, 10.1259/dmfr.20190385. PMC7333471 32155087

[29] Nishikawa K. , Kousuge Y. , and Sano T. , Is Application of a Quantitative CT Technique Helpful for Quantitative Measurement of Bone Density Using Dental Cone-beam CT?, Oral Radiology. (2016) 32, no. 1, 9–13, 10.1007/s11282-015-0202-z.

[30] Katsumata A. , Hirukawa A. , Okumura S. et al., Relationship Between Density Variability and Imaging Volume Size in Cone-Beam Computerized Tomographic Scanning of the Maxillofacial Region: an in Vitro Study, Oral Surgery, Oral Medicine, Oral Pathology, Oral Radiology & Endodontics. (2009) 107, no. 3, 420–425, 10.1016/j.tripleo.2008.05.049. 18715805

[31] Siewerdsen J. H. , Moseley D. , Bakhtiar B. , Richard S. , and Jaffray D. A. , The Influence of Antiscatter Grids on Soft-Tissue Detectability in Cone-Beam Computed Tomography With Flat-Panel Detectors: Antiscatter Grids in Cone-Beam CT, Medical Physics. (2004) 31, no. 12, 3506–3520, 10.1118/1.1819789. 15651634

[32] Ghorbanzadeh M. , Vahdat B. , Akhavanallaf A. , and Arabi H. , Comparative Study of Analytical Metal Artifact Reduction Methods in CT Imaging, 2021.

